# Internalized age stereotypes as a mediator between volunteering and psychosocial health for adults 50+

**DOI:** 10.1093/geroni/igab046.2590

**Published:** 2021-12-17

**Authors:** Andrew Steward, Leslie Hasche, Julie Anne Laser-Maira

**Affiliations:** 1 University of Denver, Lone Tree, Colorado, United States; 2 University of Denver, Denver, Colorado, United States

## Abstract

The productive aging literature describes a wide range of psychosocial benefits of volunteerism for older adults. A growing, compelling body of literature drawing from stereotype embodiment theory identifies significant, negative public health impacts of internalized age stereotypes. Yet, little research has explored which activities may reduce internalized ageism and enhance psychosocial health as people age. This cross-sectional study examined whether internalized age stereotypes mediate the relationship between volunteering and social connectedness for adults 50+. A convenience sample of volunteers (n = 112) 50+ years of age residing in the U.S. Mountain West were recruited. A 15-minute, online survey was utilized. The independent variable was number of volunteer hours per week (mean = 6.25, SD = 4.85). The dependent variable was social connectedness measured by five items positively worded from the five-point, Likert-type UCLA loneliness scale (α = .85; mean = 4.26, SD = 0.59). Drawing from the self-stereotypes of aging scale, the indirect effects of five internalized positive (e.g., “wise” and “capable”) and five negative (e.g., “grumpy” and “helpless”) age stereotypes were tested. Results indicate that increased internalized positive, not negative, age stereotypes partially mediated the relationship between volunteer hours and increased social connectedness, while holding constant age, gender, race, functional limitation, education, employment, length of volunteering, and previous volunteer experience. Although positive age stereotypes have long been considered a form of ageism, the results of this study suggest that internalizing positive age stereotypes may function as a form of esteem to promote enhanced psychosocial health as people age.

